# Effects of GH on the Aging Process in Several Organs: Mechanisms of Action

**DOI:** 10.3390/ijms23147848

**Published:** 2022-07-16

**Authors:** Jesús Á. F. Tresguerres, Isabel Fernández-Tresguerres, José Viña, Lisa Rancan, Sergio D. Paredes, Beatriz Linillos-Pradillo, Elena Vara

**Affiliations:** 1 Department of Physiology, School of Medicine, Complutense University of Madrid, Avda. Complutense, s/n, 28040 Madrid, Spain; spared01@ucm.es; 2 Department of Clinical Dental Specialties, School of Dentistry, Complutense University of Madrid, Avda. Complutense, s/n, 28040 Madrid, Spain; isabelfernandeztresguerres@odon.ucm.es; 3 Department of Physiology, School of Medicine, University of Valencia, 46010 Valencia, Spain; jose.vina@uv.es; 4 Department of Biochemistry and Molecular Biology, School of Medicine, Complutense University of Madrid, Avda. Complutense, s/n, 28040 Madrid, Spain; lisaranc@ucm.es (L.R.); beatlini@ucm.es (B.L.-P.); evaraami@ucm.es (E.V.)

**Keywords:** aging, GH, central nervous system, Bcl-2, caspases, oxidative stress, inflammation, apoptosis, TNF-alpha, NfκB

## Abstract

In order to investigate the possible beneficial effects of GH administration on the aging process, 24-month-old rats of both sexes and 10-month-old SAMP8 mice were used. Male rats showed increased fat content and decreased lean body mass together with enhanced vasoconstriction and reduced vasodilation of their aortic rings compared to young adult animals. Chronic GH treatment for 10 weeks increased lean body mass and reduced fat weight together with inducing an enhancement of the vasodilatory response by increasing eNOS and a reduction of the constrictory responses. Old SAMP8 male mice also showed insulin resistance together with a decrease in insulin production by the endocrine pancreas and a reduced expression of differentiation parameters. GH treatment decreased plasma levels and increased pancreatic production of insulin and restored differentiation parameters in these animals. Ovariectomy plus low calcium diet in rabbits induced osteoporosis Titanium implants inserted into these rabbit tibiae showed after one month lesser bone to implant (BIC) surface and bone mineral density (BMD). Local application of GH in the surgical opening was able to increase BIC in the osteoporotic group. The hippocampus of old rats showed a reduction in the number of neurons and also in neurogenesis compared to young ones, together with an increase of caspases and a reduction of Bcl-2. GH treatment was able to enhance significantly only the total number of neurons. In conclusion, GH treatment was able to show beneficial effects in old animals on all the different organs and metabolic functions studied.

## 1. Introduction

Growth hormone (GH) is the most abundant hormone present in the anterior pituitary that accounts for 4–10% of the wet weight of this endocrine gland in the human adult, amounting to about 5–10 mg per gland [1].

The circulating levels of this hormone show a decline during the first weeks after birth but reach adult levels after only two or three weeks of life. The secretion of GH is pulsatile with bursts occurring every 3–4 h over the 24 h of the day, being the secretory peaks more frequent and smaller in females than in males [2]. The most important secretion peak of GH occurs during the first two hours of nocturnal rest in the period of slow wave or deep sleep.

GH causes cells to grow and multiply by directly increasing the rate at which amino acids are used to synthesize proteins, inducing an increase in the growth rate of long bones and skeletal muscles during childhood and teenage years. GH is also an anabolic protein hormone that produces positive nitrogen and phosphorus balance [3].

In addition, GH stimulates lipolysis in the fat tissue, which is the breakdown of triglycerides into fatty acids and glycerol [4].

GH deficiency in the adult has been recognized as a specific clinical syndrome characterized by a combination of metabolic and cardiovascular features that are more evident in women than in men. The syndrome includes a high prevalence of hypertension, glucose intolerance, dyslipidemia, and central obesity. This asymptomatic situation is also associated with early arteriosclerosis. All these mentioned parameters play an important role in the increase in cardiovascular risk [5].

## 2. Physiological Decrease of GH Secretion with Age

The low GH values present in elderly people are associated with loss in bone mineral density, reduction in lean body mass and muscular strength, increase in adipose tissue, enhanced insulin resistance, and glucose intolerance, etc. [6,7,8]. GH deficiency (GHD) in adults shows similarities with the changes shown by elderly people thus pointing to a possible relationship between age-related physical impairment and the decline in GH/IGF-1 axis that occurs physiologically with age [6,7,9]. The term “somatopause” has been proposed to describe this situation [10]. Old age could be described as a physiological state of GHD. If we and other authors are supporting the original proposal of Rudman et al. 1990 [11] that the reduction of GH with age could be responsible for several alterations in the different systems and organs leading to the physical impairments associated with the elderly, then there should be the possibility that the administration of physiological amounts of this hormone could contribute to restoring, at least in part, many of those alterations, improving the quality of life in the same way as we do since the fifties of last century with the treatment of GH-deficient children.

GH treatment on the other hand, is able to enhance muscular strength, and lean body mass in humans and to reduce body fat [11,12,13], improving plasma lipid profile and increasing bone mineral density [11]. GH has also been shown to display a positive action on vascular function and structure in old individuals [14].

### The Antioxidant Effect of GH Replacement Therapy

The free radical theory of aging has provided a theoretical base to carry out experiments that contribute to a better understanding of the aging process [15]. It is now well established that the upregulation of the endogenous antioxidant defenses can be a useful mechanism for cells to prevent the damage which is associated with an excessive free radical production [16,17]. GH supplementation can act as an antioxidant because it upregulates the expression of important intracellular antioxidant enzymes, such as catalase, glutathione peroxidase, and glucose-6-phosphate dehydrogenase. Old animals treated with relatively small doses of GH suffer less oxidative stress than untreated old animals, both in terms of protein oxidation and DNA oxidation (measured in terms of levels of 8-OHdG). Supplementation with GH stimulates endogenous antioxidant enzymes, prevents oxidative damage to critical cellular structures, and thus behaves as an antioxidant. The protective effect of GH treatment on oxidative stress and inflammation has been previously demonstrated by our group in the liver, heart, and pancreas [18,19,20,21].

## 3. Metabolic Effects

It has been already reported that GH and IGF-1 production decrease with age [22]. We have reported previously that hepatic IGF-1 content is reduced in old females [23] and that GH administration was able to increase it significantly.

Old rats showed an increase in periuterine fat together with an enhancement in the total fat content of the carcasses as measured by the Specific Gravity Index (SGI). SGI establishes a correlation between lean body mass and fat mass; the higher the value, the lesser the fat is present in the animal. Under normal circumstances, at old age adiposity is augmented and lean body mass reduced. Chronic GH administration was able to reduce periuterine fat and to increase SGI in old rats both intact or castrated, which means that this treatment is able to increase muscle mass and reduce fat tissue [22]. These data are consistent with previous studies from our laboratory [24] observing the effects of GH administration on body composition. GH treatment for both GHD adults and elderly people has been shown to improve several parameters related to body composition [11,25], for example reducing waist perimeter, which has been proven to be a strong predictor of cardiovascular risk [26]. The SGI increase that was seen in our data in old GH-treated rats was also associated with an enhancement in body weight gain as compared to the weight loss observed in old untreated animals; this confirms that in old rats GH exerts a more important anabolic activity on muscles than lipolytic effects on fat tissue.

### Effect of Aging and GH Replacement Therapy on Body Composition

Although a great number of studies have been established to investigate the effects of GH supplementation on muscle mass, the controversial findings reported in the literature make it difficult to take the decision to treat or not sarcopenia with GH [27]. There are apparently contradictory findings that may be explained either by different methodological approaches or by the different GH amounts used. When high doses of GH are used, a high incidence of adverse effects can be seen [12,28]. On the contrary when physiological doses of GH are employed, which are able to restore in old animals’ plasma levels of GH and IGF-1 similar or only slightly above those present in the young ones, the positive actions are maintained, but the negative side effects are highly reduced. So, in our experience GH replacement therapy has been shown to be useful in the prevention of age-related muscle mass loss [29].

Old animals showed an increase in fat content and a reduction in lean body mass that was reverted when treated with GH for two months and a half.

**Figure 1:**
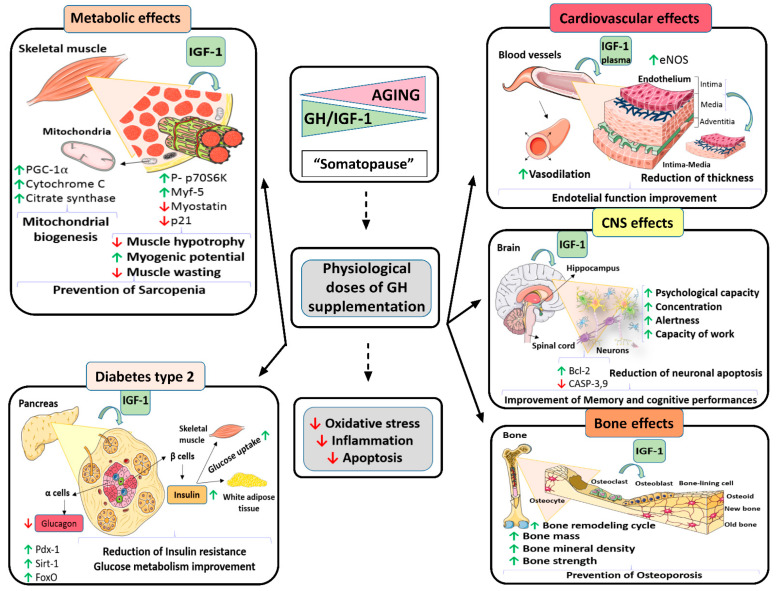
Beneficial effects of chronic treatment with physiological doses of GH in both old Wistar rats or senescence prone SAMP8 mice on all the different organs and functions studied. Figure created with Smart Servier Medical Art.

Muscle protein synthesis has been shown to decrease with age [30] whereas protein degradation shows an increase. Skeletal muscle hypertrophy needs p70S6K as has been already seen in various animal models [31]. When activated, mTOR plays a role in translation initiation by phosphorylating p70S6K, which, in turn, also phosphorylates S6 ribosomal protein allowing the upregulation of a subclass of mRNAs encoding the translational apparatus [32]. Skeletal muscles from old rats show a lower basal phosphorylation of p70S6K than in muscles from young animals and this was not due to changes in p70S6K protein levels. Treatment with GH in old animals was able to restore phospho-p70S6K reaching the values found in young animals (Figure 1). 

The observed attenuation in the capacity for hypertrophy in muscles of old individuals has also been associated with an age-related impairment in its myogenic potential [33]. In order to study this process, the myogenic response of gastrocnemius muscle to a GH treatment in young and old rats was investigated. Repair or regeneration and growth of mature myofibers [34] need Myf-5 as a stimulator for myoblast/satellite cell differentiation. GH treatment has been shown to upregulate skeletal muscle IGF-1 gene expression [35] that plays a role in the activation of satellite cells [36]. Although no decrease in Myf-5 skeletal muscle protein levels during aging was observed, GH replacement therapy significantly increased the values of this myogenic factor (Figure 1). 

On the other hand, myostatin, a factor associated with the TGFβ family, plays a negative regulatory role on muscle [36]. The function of this factor, whose expression is restricted to muscle tissue [37], is to keep the satellite cells in the quiescent phase through the induction of p21 [36]. In the absence of myostatin or when its action is blocked, a massive skeletal muscle hypertrophy takes place that has been attributed to the proliferation of muscle fiber-associated satellite cells [38]. Significant increases both in myostatin and p21 in aged muscles have been previously found [39]. However, GH replacement therapy has been able to reduce both significantly, thus contributing to the prevention of muscle wasting (Figure 1). 

Mitochondrial content and activity are heavily reduced with age [40,41]. PGC-1α is a molecule that responds to changes in oxidative stress [16,42] and also plays a master role in mitochondrial biogenesis [42,43]. Since aging in muscle as in other tissues is associated with an increase in oxidative stress parameters, we have seen that aging also resulted in a decrease in PGC-1α expression in muscle. Treatment with relatively low doses of GH was able to prevent oxidative stress in muscle and thus the aging-associated PGC-1α decrease. Cytochrome C protein levels and citrate synthase are two markers of mitochondrial activity that were found to be also lower in the muscle of old animals, but this situation was completely prevented when animals were treated with GH. Exercise activation of PGC-1α does not take place when animals are old [44]. This absence of response may be due to a blockade of the activating mechanisms that under normal circumstances are induced by endogenous GH. However, when this hormone is exogenously given to animals, PGC-1α is activated as well as mitochondrial biogenesis. The ability of GH supplementation to maintain normal muscle levels in the old animals could be supported by our previous data showing the antioxidant effects discussed previously and that PGC-1α could also be activated by MAP kinases. In a previous paper from our group [44], it was shown that neither cold exposure, exercise, nor even thyroid hormone treatment could activate PGC-1α in the old animals. The activation of PGC-1α by exogenous administration of GH that has been reported in this study seems to be unique in maintaining normal muscle mass in the old animals and thus preventing sarcopenia.

We would like to insist on the fact that the GH supplementation that we have given to the old animals is not very high, aiming to obtain a return in the physiological levels observed in young animals. Although supplementation with high doses of GH should not be recommended, small or physiological doses of GH supplementation could be a useful way to prevent age-associated sarcopenia.

## 4. Cardiovascular Effects

GH-deficient patients show an increased cardiovascular risk [45] with endothelial dysfunction, including a reduced vascular endothelial-dependent relaxation [46].

On the other hand, aging is associated with both structural and functional changes in the vascular wall [47]. For example, an increase in media-intima thickness has been detected together with changes in cellular and extracellular composition of the vessel wall [48]. Aging in rats has been also associated with reduced endothelium-dependent vasodilatation. Similar results have been obtained in humans, when measuring the vasodilatory response to brachial artery infusion of acetylcholine or to ischemia by plethysmography [49].

The important mechanism underlying the altered response to endothelium-dependent agents during aging seems to be a decrease in endothelial NO availability due to reduced synthesis and/or major degradation by oxidative stress [47,48] which has been confirmed by our group [20,50]. In addition, this altered endothelial function may also involve an increase in contracting factors which could counteract the effect of the relaxing ones. In previous studies from our group, the chronic exogenous administration of GH for two months to old rats induced an expected increase in plasma levels of IGF-1 that was accompanied by an improvement of endothelial function and vessel structure [51,52] leading to an increase in relaxation and a reduction of vasoconstriction (Figure 1). 

The beneficial effects exerted by GH involved an increase in endothelial NO availability that has been confirmed by our group [20,50]. These data confirm previous studies which show that GH can exert beneficial effects on cardiovascular function in aged animals [53,54].

## 5. Type 2 Diabetes

The senescence-accelerated mice (SAM) models include two types of animals: senescence-accelerated prone mice (SAMP, short-living) and senescence-accelerated-resistant mice (SAMR, longer living), which can serve as their controls [55]. These mice may show different phenotypes, including alterations in learning and memory, impaired immune response, and abnormal circadian rhythm [55]. SAMP8 is a substrain of SAMP, which has been extensively used in several studies and shows many of the typical alterations of the SAMP aging phenotype. It is normally compared with SAMR1 animals, which can serve as their controls [56,57] related to diseases, etc. [58,59]. SAMP8 mice have been used in studies related to vascular and cardiological functions and also to neurodegenerative diseases, etc., and have been found to be a suitable model for the study of aging [56,60]. Since animals are already old at 10 months of age and have a suitable control group, this fact includes several advantages. Approximately, the median survival time of SAMP mice is 9.7 months, 40% shorter than that of the SAMR strains (16.3 months) [55]. There are other studies showing that SAMP8 median life span can last until 17.2 months [60] and SAMR1 mice as long as 21 months of age [61].

SAMP8 mice at 10 months of age show increased insulin resistance with elevated plasma insulin levels together with a reduced content in the endocrine pancreas due to the exhaustion of β-cells by the maintained increased synthesis. These findings were found together with the maintenance of normoglycemia as compared with young mice since the increased plasma levels were still allowing the compensation to the insulin resistance. It is well known that hyperglycemia appears only when the β-cells are not more able to maintain this compensatory insulin over-secretion. The appearance of a β-cell inability to compensate for the increased resistance takes place when several circumstances occur, including elevated glucose-induced oxidative stress and inflammation in the endocrine pancreas, leading to cellular damage and apoptosis [62,63,64]. Cellular models of glucose toxicity show reduced Pdx-1 expression in the pancreas, which is normally associated with the development of type 2 diabetes. A correlation has been found between the reduction of Pdx-1 levels and the failure of β-cells [65,66,67,68,69]. Old SAMP8 mice also showed reduced expression of Pdx-1 mRNA in the pancreas as compared to young animals [70].

Type 2 diabetes not only shows a reduced β-cell mass and function, but also a relative increase and dysfunction of α-cells leading to hyperglucagonemia [71,72,73]. Our data also show an increase in glucagon mRNA expression in old SAMP8 mice together with an increase in GLUT2 mRNA expression [74]. The reduced expressions of FoxO factors and IGF-1 have been also observed in old SAMP8 mice, whereas in the control animals (SAMR1 animals) no significant differences were detected. Sirtuin 1 is a factor involved in the support of the expression of FoxO1 target genes that promotes the expression of insulin gene transcription elements [75].

On the other hand, IGF-1 is the GH effect mediator and plays a role in islet cell growth, in the maintenance of insulin secretion and sensitivity. A decrease in mRNA expression of IGF-1 in the pancreas of old animals has been observed whereas the chronic treatment with GH was able to restore these levels reaching similar values to those found in the young animals.

Forkhead box O (FoxO) transcription factors play an important role in modulating several metabolic functions [68] according to the nutritional situation. When nutrients and insulin levels are low, FoxO promotes gluconeogenesis. However, in the presence of insulin resistance, the negative signaling of FoxO1 is compromised [76]. In old senescence prone mice chronic GH replacement therapy was able to increase mRNA expression of FoxO1 and also of sirtuin 1. Since FoxO1 may preserve β-cell function on one hand but its activity also prevents on the other the increase in β-cell mass, it can be suggested that mRNA expression of proliferative genes should be decreased in old SAMP8 animals. However, no significant differences were detected in the mRNA expression of proliferative genes in the pancreas (PCNA and Sei1) either with aging or with GH treatment. On the other hand, GH treatment induced a Pdx-1 increase of the age-related lower values. The major hormones controlling metabolism include glucagon, insulin, adrenal glucocorticoid hormone, corticosterone, and, potentially, somatostatin [68]. The mRNA expression of glucagon was elevated with aging and treatment with GH was able to reduce it. Furthermore, GH treatment was able to decrease plasma levels of insulin in old male SAMP8 mice thus leading to a decrease in HOMA-IR index in these animals, which was elevated due to those age-related high plasma levels. When the animals were treated with exogenous GH, the requirements for insulin were lower, leading to a reduction in plasma insulin levels and, also, to a decrease in the expression of the glucagon gene.

Pancreatic β-cells of the Langerhans islets play a very important role in glucose homeostasis in mammals maintaining physiological plasma levels by secreting insulin in response to the elevation in blood glucose levels observed after carbohydrate absorption after a meal. The highly coordinated mechanism senses plasma glucose using GLUT2 (glucose transporter 2) in the β-cell and at the same time, glucose enters the cell and is phosphorylated resulting in an increase in ATP which in turn closes a K^+^ channel that depolarizes the cell and later opens a Ca^2+^ channel that induces the secretion of the insulin granules [73,74].

GLUT2 expression showed an increase with aging and GH treatment was able to reduce it. The observed reduction in GLUT2 mRNA expression could be due to the recovery of a normal insulin sensitivity in several peripheral tissues like muscle or fat so that the increase in plasma levels was not any more needed.

Our results have shown that GH administration to old SAMP8 mice was able to improve their endocrine pancreatic function thus decreasing insulin and glucagon expressions in the islet cells and improving glucose metabolism [77], confirming other results showing a positive action on islet mass in streptozotocin-induced diabetes [78] (Figure 1). 

## 6. Effects on Bone

Although dental implants are a very good alternative for prosthetic rehabilitation, under certain circumstances implant therapy is a real challenge for clinicians when the patients suffer from atrophic maxillae or severe osteoporosis [79]. It is well known that age is associated with a decrease in bone mass and also with the deterioration of bone microarchitecture [80]. This is similar to what happens in osteoporosis. Osseointegration of the implants needs a physiologically reacting bone tissue and when those are porotic it is usually very difficult to achieve [81,82].

The bone is continuously undergoing a remodeling process of the tissue that includes resorption by osteoclasts and new bone formation by osteoblasts. The remodeling process under normal conditions is balanced, but when osteoporosis or aging are present, a displacement towards bone resorption can be observed. GH administration together with other hormones like estrogens or parathormone [83] has been shown to increase bone mass in the elderly [79,80,81,82] and has also been able to enhance bone mass in osteoporosis [83].

GH administration in vivo is a potent direct stimulator of bone osteoblast number and function and is also able to act, through the release of the mediator insulin-like growth factor 1 (IGF-1), from several organs. When treated with GH, osteoblasts are able to synthesize IGF-1 stimulating directly collagen synthesis [84]. GH treatments have shown benefits on bone metabolism in humans, increasing bone mineral density and bone strength in patients with growth hormone deficiency and osteoporosis [85,86,87].

GH when given systemically is also able to stimulate the synthesis of bone proteins and their mineralization [88], thus increasing bone turnover [89], and speeding up bone fracture repair, both in young and old animals [89]. Thus, GH is a very important anabolic factor for the bone. However, although recognized as a local growth factor [90,91] only very few studies have demonstrated direct effects of GH when locally administered [92].

Some time ago previous studies from our group demonstrated for the first time that the local application of GH into the ostectomy performed previously to the insertion of a titanium sheet into the tibia of an osteoporotic rabbit was able to induce a great local peri-implant reaction [92]. Later on, and in order to evaluate the bone to implant contact, GH was given to investigate if it could be able to induce a significant effect in the bone-to-implant contact (BIC) comparing GH-treated and untreated groups, both in osteoporotic and non-osteoporotic rabbits. BMD was also evaluated using dual-energy X-ray absorptiometry (DXA) around the implant [93].

GH is apparently the only hormone that is able to stimulate both the proliferation and differentiation of osteoblast [94] and also osteoclast differentiation [95], thus it should be able to stimulate the whole bone remodeling process and to increase bone mass in vivo [91]. This has been proven in GH-deficient patients, by the improvement in bone matrix protein synthesis and mineralization [96,97]. The process followed the physiological pathway of first stimulating bone resorption and then new bone formation [98].

Ovariectomized animals treated with GH in the ostectomy before the insertion of the implant showed the highest BIC value (36.16 ± 9.86), being even higher than those found in intact animals (34.89 ± 4.69). Locally applied, GH was able to accelerate the osseointegration process, even under very low calcium conditions [99] (Figure 1). 

Higher BIC values in healthy rabbits, 2 and 6 weeks after implant insertion were also observed in other studies of our group [100,101,102]. Gomez-Moreno et al. [103] found that local application of 4 IU of GH was able to statistically increase BIC and peri-implant bone area 2 weeks after surgery in non-osteoporotic Beagle dogs, results that were quite similar to ours.

BMD around implants in the intact group treated with GH was investigated using densitometry and showed no significantly lower values than in the intact control group. This was in agreement with previous experiments [101] that demonstrated a non-statistically significant reduction in BMD 2, 3, and 6 weeks after surgery in healthy rabbits when treated locally with GH. The observed decrease in BMD could be explained by the GH acceleration in bone remodeling, augmenting the number of bone replacement units and its space in the cortical, making it more porous and reducing the BMD. This phenomenon must be taken into account when systemic GH is used for the treatment of osteoporotic bones, since a net gain in bone mass can only be found when GH treatment is prolonged in time. Actually, in humans, it is necessary to wait for a minimum of 18 months of treatment to obtain a real increase in bone mass [104,105].

## 7. Effects on Central Nervous System

GH has also been shown to act on the central nervous system (CNS) [106]. GH deficiency is associated with several cognitive impairments, memory loss, sleep disturbances, and a feeling of reduced wellbeing. GH replacement therapy has been shown to be able to reduce alterations in memory and cognitive performances in GH-deficient patients [107,108,109,110,111,112,113,114].

GH administration was also able to protect the brain and the spinal cord from different forms of neurodegenerative stimuli in animal models and to prevent the neuronal death that occurs after hypoxic-ischemic injury [115,116,117]. The decrease that can be detected in GH levels with age [118] may affect its neuroprotective effect on the brain and may contribute to the deterioration of its function that is associated with age [119,120,121,122].

Neurogenesis is maintained through adult life in the dentate gyrus of rodents, but cell proliferation in this area of the brain gets dramatically decreased with age [123].

The brain is one among the many tissues where GH carries out its effects [106] leading in adults to an increase in memory, concentration, alertness, psychological capacity [110], and capacity for work. There are GH receptors in the different cells that form the CNS, endothelial cells in the blood vessels glia, and of course neurons [124]. GH stimulates the production of IGF-1 in the CNS [125] playing most probably a trophic role locally [126]. Young animals show the production of new neurons (neurogenesis) in several areas of the brain although its real significance is still unknown [126,127].

A significant sex dimorphism in total neuronal content in the hilus of the dentate gyrus of the rat hippocampus has been detected. All animals show in both sexes a preservation of the neuronal content until 22 months of age, followed by a significant neuronal loss until 24 months of age [128]. The number of hilar neurons is also sexually dimorphic in the rat, with more neurons present in males than in females. The difference has been detected in young animals and is maintained until 24 months of age. However, the hilus of both sexes gets a reduction in neurons between 22 and 24 months of age. These results are also similar to a previous report showing the same neuronal loss in the hilus of 24-month-old male Fischer 344 rats when compared to 12-month-old animals [128].

If we carry out a treatment with GH for 10 weeks, the total number of hilar neurons in 24-month-old rats is higher than in vehicle-treated age-matched controls (Figure 1). GH administration in young animals has been shown to be a neuroprotective factor for the brain and spinal cord [129,130,131]. GH administration to old rats is able to increase IGF-1 expression in the hippocampus [126] and other areas in the brain [127]. IGF-1 has shown neuroprotective actions in hilar neurons [132,133,134], so that the increased local production of this factor may play a relevant role in the neuroprotective effects of GH. Our data indicate that GH plays a protective role on hilar hippocampal neurons preventing age-related apoptosis [129,130]. This neuroprotective effect of GH has been observed both in males and in females.

The increased number of nucleosomes (apoptosis markers) in aging brain homogenates together with the reduction in Bcl-2, indicated the presence of an age-related apoptosis increase.

This nucleosome increase was also associated with an enhancement of proapoptotic caspases 3 and 9. Chronic GH treatment induces a clear reduction of apoptosis that was accompanied by an increase in Bcl-2 levels and a decrease in nucleosomes. These changes could be due, either to GH directly or by its activation of IGF-1, whose levels are also increased after the subcutaneous GH injection [128,135]. A possible mechanism could be the inhibition exerted by GH and/or IGF-1 on Pl3-kinase, mediating the phosphorylation of Akt/protein-kinase (Akt/PKB) [136] leading to the phosphorylation of Bad [133], making it unable to bind anti-apoptotic proteins like Bcl-2, and allowing this protein to increase. In addition, Akt/PKB activates the transcription factor CREB contributing to the stimulation of Bcl-2 expression. The Bcl-2 elevation could be responsible, first, for a decrease in caspase-9, and also, for the caspase-3 decrease. A survival factor like sirtuin 1 and an antioxidant like glutathione peroxidase showed increases in the GH-treated animals thus contributing also to the reduction in apoptosis [129,130].

The dentate gyrus of all studied animals showed cells with BrdU-positive nuclei that were found in the subgranular zone (SGZ) in the inner part of the granule cell layer (GCL) and in the hilus. BrdU-immunoreactive cell number in the GCL and SGZ of the dentate gyrus was drastically reduced by aging and the treatment with GH was not able to increase its numbers, so the observed increase in the total number of neurons was more due to the reduction in apoptosis than to an increase in neurogenesis [128].

However, if GH is applied after a brain lesion it is able to potentiate neurogenesis allowing a rapid healing of the brain damage [117] as has been demonstrated also in humans by Devesa et al. [137,138], making the treatment with GH a very important and interesting procedure for several lesions in the CNS.

## 8. Conclusions

GH levels are reduced with aging and this reduction could be responsible for several of the age-related alterations. Chronic GH replacement therapy was able to show beneficial effects in those age-related alterations that were evident on all the different organs and metabolic functions studied both in old Wistar rats or senescence-prone SAMP8 mice. These included modifications in body composition, increasing lean body mass and reducing fat content, improvements in cardiovascular function, reduction in insulin resistance improving glucose metabolism, potentiation of osseointegration, and increasing the number of neurons present in the hippocampus without affecting neurogenesis. These positive effects open new possibilities for the use of GH in old age.
